# Managing COVID-19 care in the pandemic context: impact of family health coverage

**DOI:** 10.1590/0034-7167-2024-0620

**Published:** 2025-12-08

**Authors:** Ana Paula de Vechi Corrêa, Rodrigo das Neves Cano, Gustavo Diego Magno, Isabel María López-Medina, Carmen Álvarez-Nieto, Sílvia Carla da Silva André Uehara

**Affiliations:** I Universidade Federal de São Carlos. São Carlos, São Paulo, Brazil; II Universidad de Jaén. Jaén, Spain

**Keywords:** COVID-19, Primary Health Care, Public Health Surveillance, Health Management, National Health Strategies., COVID-19, Atención Primaria de Salud, Vigilancia en Salud Pública, Gestión en Salud, Estrategias de Salud Nacionales.

## Abstract

**Objectives:**

to compare the management of Primary Health Care services in tackling the COVID-19 pandemic among Brazilian municipalities, considering Family Health team coverage.

**Methods:**

a descriptive cross-sectional study conducted with 1,134 municipal health managers. Data were collected using Google Forms^®^ and analyzed using Prevalence Ratios using a Poisson regression model with random effects.

**Results:**

municipalities with team coverage of less than 25% showed greater readaptation of services and restructuring of Primary Health Care flow (7%; CI: 1; 1.14), coverage between 25%, and 49.99% of teams showed greater restructuring of Primary Health Care flow (7%; CI: 1.02; 1.12) and changes in referral and counter-referral services (21%; CI: 1.05; 1.38).

**Conclusions:**

the teams reorganized and restructured services during the critical period of the pandemic, strengthening family health as the main service of Primary Health Care.

## RESUMEN

**Objetivos:**

comparar la gestión de los servicios de Atención Primaria de Salud en la respuesta a la pandemia de COVID-19 entre municipios brasileños, considerando la cobertura de los equipos de Salud Familiar.

**Métodos:**

estudio descriptivo transversal realizado con 1134 gestores municipales de salud. Los datos se recopilaron mediante *Google Forms* ^®^ y se analizaron mediante razones de prevalencia mediante un modelo de regresión de Poisson con efectos aleatorios.

**Resultados:**

los municipios con cobertura de equipos inferior al 25% mostraron una mayor readaptación de los servicios y reestructuración del flujo de Atención Primaria de Salud (7%;IC:1;1,14); los municipios con cobertura entre el 25%, y el 49,99% de los equipos mostraron una mayor reestructuración del flujo de Atención Primaria de Salud (7%;IC:1,02;1,12) y cambios en los servicios de referencia y contrarreferencia (21%;IC:1,05;1,38).

**Conclusiones:**

los equipos reorganizaron y reestructuraron los servicios durante el período crítico de la pandemia, fortaleciendo la salud familiar como el principal servicio de la Atención Primaria de Salud.

## INTRODUCTION

The critical phase of the pandemic has imposed new challenges on health systems worldwide due to its high transmissibility, little knowledge about treatments, and effective prevention measures and medications, in addition to the challenging start of vaccination with the insufficiency of immunobiological agents and an unprepared logistics system in many countries^(1)^.

In an unprecedented scenario in the modern history of public health, the COVID-19 pandemic required agile and effective adaptation in health systems, overcoming obstacles related to resource allocation, bed management, protocol development, among other challenges. In this context, health managers’ work became essential in coordinating services, making evidence-based decisions, and implementing measures aimed at mitigating the impact of the disease on the community, in addition to seeking to maintain the provision of quality care for chronic conditions and common problems in Primary Health Care (PHC)^(2)^.

It is undeniable that the pandemic also created learning opportunities at various levels of management, enhancing the integration of healthcare services and Health Care Networks (In Portuguese, *Rede de Atenção à Saúde* - RAS), coordinated by PHC, in addition to improving the culture of risk management, establishing educational actions among health workers and the community, incorporating technologies into health work routines, and political recognition, in order to guarantee financial resources and strengthen the health system in the medium and long term^(3)^.

One of the major challenges for primary care during the pandemic was maintaining the monitoring of people with chronic diseases, coupled with the new scenario imposed by COVID-19 on the reorganization of health units and the local health system itself. In the United States of America, a study revealed that primary care services faced greater challenges in monitoring patients with chronic diseases, highlighting vulnerabilities in the health system due to disruption of work processes during the pandemic^(4)^.

In Canada, the importance of integration between PHC and RAS was highlighted, with fluid communication among parties, with a view to building a care plan, while also suggesting the need to leverage a care model aimed at integrating systems, providing governance arrangements that guarantee this integration^(5)^.

Furthermore, the response to the pandemic varied significantly across countries. Examples include South Korea, India, Turkey, and China, which opted for a governance-based approach with strong state intervention, while others, such as Brazil, Portugal, and Switzerland, sought to strengthen preventive care models and incorporate telemedicine^(6)^. These different approaches and experiences reinforce the importance of adaptation and continuous learning in facing health emergencies and in the search for effective solutions in providing healthcare to the community^(4)^.

It is worth noting that, in Brazil, the Family Health Strategy (FHS) is the main PHC policy, providing healthcare with a community focus. During the critical phase of the pandemic, it promoted the reorganization of services, in order to guarantee the promotion of quality of life and the protection of the assisted population^(7)^. Furthermore, the FHS distribution is uneven in Brazil, despite having FHS coverage of 62.6% of Brazilians in 2019. It is noteworthy that the Northeast and South regions have the highest FHS coverage. On the other hand, the Southeast region has the highest absolute number of registered Brazilians, reflecting the greater population concentration^(8)^.

However, it is necessary to consider that Brazil, due to its territorial extension, presents inequalities in living conditions that condition people to exposure to health risks and problems, as well as unequal access to healthcare services, in addition to presenting a greater concentration of goods and services in more economically developed urban centers^(9)^.

Taking the critical phase of the COVID-19 pandemic as a starting point, it becomes clear that PHC performance was affected during this period, requiring management effort and work to adapt these services^(10)^. However, there remains a gap in knowledge, especially regarding how a country of continental dimensions reorganized PHC to face the critical phase of the pandemic, considering the management of services in different regions of the country and the influence of Family Health team (FHt) coverage in this process.

## OBJECTIVES

To compare the management of PHC healthcare services in tackling the COVID-19 pandemic among Brazilian municipalities, considering FHt coverage.

## METHODS

### Ethical aspects

The research was approved by the *Universidade Federal de São Carlos* Research Ethics Committee. Informed Consent Form was obtained online from all individuals involved in the study.

### Study design, period and location

This is a cross-sectional, descriptive study, guided by STrengthening the Reporting of OBservational studies in Epidemiology.

The target population consisted of PHC managers from Brazilian municipalities that had confirmed at least one case of COVID-19 between February 26, 2020, and June 30, 2021. A sample size calculation was performed, considering one manager per municipality, using a relative error of 5.82%, assuming a prevalence of 50% and a 95% Confidence Interval. The sample size was defined as 1,134 participants.

Primary care managers in a municipality who had worked for at least three months during the health emergency imposed by the COVID-19 pandemic were included in the study. Managers who were on leave and/or vacation during the pandemic were excluded.

### Study protocol

Data were collected through a self-administered questionnaire by PHC service managers, using Google Forms^®^, between April and September 2022. This questionnaire was constructed based on the Ministry of Health’s coronavirus (COVID-19) clinical management protocol in PHC^(11)^, containing the following variables: identification of suspected cases of influenza-like illness and COVID-19; measures to prevent contagion in health units; stratification of the severity of influenza-like illness; therapeutic management and home isolation of mild cases; early diagnosis and referral to emergency or hospital services for severe cases; immediate notification; clinical monitoring; community prevention measures; and support for active surveillance.

The questionnaires were emailed to municipal managers. To increase participation, the survey was publicized by the Brazilian National Council of Health Departments and the Brazilian National Council of Municipal Health Departments, which emphasized the importance of municipal participation and forwarded the instrument to municipal health departments. Supporters of Councils of Municipal Health Departments also collaborated in disseminating the survey to Regional Health Departments.

### Analysis of results and statistics

For FHt coverage analysis, no ideal parameter for these services was found in the literature. Therefore, the researchers defined the following classification: <25%; from 25.01% to 49.99%; from 50% to 74.99%; and >75%. The FHt coverage of the participating municipalities was taken from the Ministry of Health’s e-Gestor AB website^(12)^.

Absolute and relative frequencies were used to describe qualitative variables. To estimate Prevalence Ratios, comparing the FHt’s coverage range, a Poisson regression model with random effects was used^(13)^. All analyses were performed using SAS 9.4 software. A significance level of 5% was adopted for all analyses.

## RESULTS

A total of 1,134 managers from all over the country participated in the research, 40.4% (458) from the Southeast, 27.9% (316) from the Northeast, 18.6% (211) from the South, 8.5% (97) from the North and 4.6% (52) from the Central-West. Of these, 79.4% (900) reported being female and 20.6% (234) were male, with an average age of 39.7 years. Concerning FHt coverage, it is noteworthy that coverage greater than 75% was present in 896 (79%) participating municipalities.

In comparisons between FHt coverage and the reorganization of PHC services to combat the pandemic, all participants responded that PHC services were adapted to address COVID-19. In this context, it was evident that the prevalence of service readaptation and restructuring of PHC flow was 7% (95%CI: 1; 1.14) higher in municipalities with FHt coverage below 25% when compared to municipalities with coverage above 75%. Furthermore, the reporting of suspected/confirmed COVID-19 cases within 24 hours was 1% (1; 1.02) higher in municipalities with FHt coverage below 25% when compared to municipalities with coverage above 75% (Table 1).

**Table 1:** Organization of Primary Health Care management in tackling the COVID-19 pandemic, considering Family Health team coverage <25% versus between 25% and 49.99%; <25% versus between 50% and 74.99%; and <25% versus >75%

Variable	<25% versus between 25% and 49.99%		<25% versus between 50% and 74.99%		<25% *versus* ≥75%	
	PR (95%CI)	*p* value	PR (95%CI)	*p* value	PR (95%CI)	*p* value
Have PHC services been adapted to address COVID-19?						
No	-	-	-	-	-	-
Yes	1.03 (0.96; 1.11)	0.43	0.99 (0.93; 1.05)	0.72	1.07 (1; 1.14)	0.04
Has PHC workflow been restructured during the COVID-19 pandemic?						
No	-	-	-	-	-	-
Yes	1.01 (0.94; 1.08)	0.88	1.03 (0.96; 1.1)	0.48	1.07 (1.01; 1.14)	0.02
Have PHC referral and counter-referral services within RAS changed during the COVID-19 pandemic?						
No	-	-	-	-	-	-
Yes	1.03 (0.79; 1.33)	0.85	1.04 (0.79; 1.38)	0.76	1.24 (0.97; 1.58)	0.09
Frequency Missing = 1						
Has the physical structure of PHC services been adapted to handle suspected and confirmed cases of COVID-19?						
No	-	-	-	-	-	-
Yes	1.16 (0.87; 1.53)	0.31	0.9 (0.69; 1.18)	0.46	1.15 (0.89; 1.48)	0.30
Have healthcare professionals considered at risk for developing severe COVID-19 been assigned to work from home?						
No	-	-	-	-	-	-
Yes	0.82 (0.65; 1.03)	0.08	0.8 (0.64; 1)	0.05	0.8 (0.64; 1)	0.05
Are suspected cases of COVID-19, when received by health units, reported by healthcare professionals within 24 hours?						
No	-	-	-	-	-	-
Yes	1.01 (0.99; 1.03)	0.33	1 (1; 1)	.	1.01 (1; 1.02)	<0.01
Are COVID-19 cases reported in RAS previously assessed by PHC?						
No	-	-	-	-	-	-
Yes	1.05 (0.88; 1.24)	0.61	1 (0.87; 1.15)	0.97	0.96 (0.85; 1.08)	0.50
Frequency Missing = 1						
Have PHC services implemented specific protocols to combat COVID-19?						
No	-	-	-	-	-	-
Yes	1.04 (0.93; 1.15)	0.51	1 (0.9; 1.1)	0.92	1 (0.92; 1.09)	0.98
Have PHC healthcare professionals received any training to care for suspected cases of COVID-19 and manage users diagnosed with COVID-19?						
No	-	-	-	-	-	-
Yes	1.01 (0.92; 1.12)	0.83	1.07 (0.96; 1.21)	0.22	1.05 (0.96; 1.15)	0.28
Did healthcare professionals receive adequate PPE?						
No	-	-	-	-	-	-
Yes	1.03 (0.99; 1.08)	0.10	1.01 (0.99; 1.03)	0.31	1.01 (1; 1.02)	<0.01
Were healthcare professionals provided with adequate quality PPE?						
No	-	-	-	-	-	-
Yes	1.01 (0.99; 1.03)	0.33	1.03 (1; 1.06)	0.06	1.01 (1; 1.02)	<0.01
Were users of the health unit, when presenting flu-like symptoms with saturation levels lower than 95% during spontaneous breathing, in addition to presenting some comorbidity that puts them at risk for worsening COVID-19, referred to other RAS points?						
No	-	-	-	-	-	-
Yes	0.92 (0.83; 1.03)	0.14	0.96 (0.85; 1.07)	0.44	0.94 (0.84; 1.04)	0.21
Was information technology used for telecare in pre-clinical care, diagnostics, case monitoring and consultations?						
No	-	-	-	-	-	-
Yes	0.92 (0.7; 1.22)	0.58	1.03 (0.78; 1.37)	0.85	1.03 (0.8; 1.32)	0.83
Did users over 60 years of age, those with immunosuppression, chronic illnesses, pregnant women, and women in the postpartum period receive priority care when entering the health unit reporting signs and symptoms of flu-like illness?						
No	-	-	-	-	-	-
Yes	1.03 (1; 1.07)	0.05	1.03 (1; 1.06)	0.08	1.02 (1.01; 1.03)	<0.01
Was monitoring of users considered at risk carried out via telephone every 24 hours?						
No	-	-	-	-	-	-
Yes	1.01 (0.81; 1.25)	0.93	0.98 (0.79; 1.2)	0.83	0.88 (0.73; 1.05)	0.17
Was monitoring of users with flu-like symptoms carried out via telephone every 48 hours?						
No	-	-	-	-	-	-
Yes	0.93 (0.75; 1.17)	0.55	0.81 (0.66; 1)	0.05	0.81 (0.67; 0.99)	0.04

*PHC - Primary Health Care; FHt - Family Health Team; PR - Prevalence Ratio; 95%CI - 95% Confidence Interval; p-value - significance level; RAS - Health Care Network; PPE - Personal Protective Equipment.*

Regarding Personal Protective Equipment (PPE), the availability of adequate quantity and quality of PPE was 1% (CI: 1; 1.02) more prevalent in municipalities with FHt coverage of less than 25% when compared to municipalities with coverage greater than 75%. A similar fact was found in the prioritization of care for groups at risk for worsening of COVID-19, which was also 2% (CI: 1.01; 1.03) more prevalent in municipalities with FHt coverage of less than 25% when compared to municipalities with coverage greater than 75% (Table 1).

It is important to highlight that, although the results showed a statistically significant difference, when assessed in practice, these values of 1% and 2% do not have an epidemiological impact.

Monitoring of COVID-19 patients by telephone every 48 hours was 19% (CI: 0.67; 0.99) less prevalent in municipalities with FHt coverage of less than 25% when compared to municipalities with coverage greater than 75% (Table 1).

In municipalities with FHt coverage between 50% and 74.99%, the adaptation of PHC services to cope with the pandemic was 8% (95%CI: 1.04; 1.13) more prevalent than in those with coverage greater than 75%. For restructuring PHC flow during the pandemic, the prevalence was 7% (95%CI: 1.02; 1.12) higher in municipalities with FHt coverage between 25% and 49.99% than in those with coverage greater than 75%. Changes in PHC referral and counter-referral services within RAS were 21% (95%CI: 1.05; 1.38) more prevalent in municipalities with FHt coverage between 25% and 49.99% when compared to municipalities with coverage greater than 75% (Table 2).

**Table 2:** Organization of Primary Health Care management in tackling the COVID-19 pandemic, considering Family Health team coverage between 25% and 49.99% versus between 50% and 74.99%; between 25% and 49.99% versus >**75%; between 50% and 74.99% versus** >75%

Variable	Between 25% and 49.99% versus between 50% and 74.99%		Between 25% and 49.99% versus ≥75%		Between 50% and 74.99% versus ≥75%	
	PR (95%CI)	*p* value	PR (95%CI)	*p* value	PR (95%CI)	*p* value
Has PHC workflow been restructured during the COVID-19 pandemic?						
No	-	-	-	-	-	-
Yes	0.96 (0.91; 1.01)	0.11	1.04 (0.99; 1.1)	0.14	1.08 (1.04; 1.13)	<0.01
Has PHC workflow been restructured during the COVID-19 pandemic?						
No	-	-	-	-	-	-
Yes	1.02 (0.96; 1.08)	0.51	1.07 (1.02; 1.12)	<0.01	1.05 (0.99; 1.1)	0.08
Have PHC referral and counter-referral services within RAS changed during the COVID-19 pandemic?						
No	-	-	-	-	-	-
Yes	1.02 (0.84; 1.23)	0.85	1.21 (1.05; 1.38)	<0.01	1.18 (1; 1.4)	0.05
Frequency Missing = 1						
Has the physical structure of PHC services been adapted to handle suspected and confirmed cases of COVID-19?						
No	-	-	-	-	-	-
Yes	0.78 (0.66; 0.92)	<0.01	0.99 (0.85; 1.15)	0.92	1.27 (1.13; 1.42)	<0.01
Have healthcare professionals considered at risk for developing severe COVID-19 been assigned to work from home?						
No	-	-	-	-	-	-
Yes	0.98 (0.88; 1.09)	0.69	0.98 (0.91; 1.07)	0.70	1 (0.94; 1.08)	0.90
Are suspected cases of COVID-19, when received by health units, reported by healthcare professionals within 24 hours?						
No	-	-	-	-	-	-
Yes	0.99 (0.97; 1.01)	0.33	1 (0.98; 1.02)	1.00	1.01 (1; 1.02)	<0.01
Are COVID-19 cases reported in RAS previously assessed by PHC?						
No	-	-	-	-	-	-
Yes	0.95 (0.83; 1.09)	0.50	0.92 (0.81; 1.03)	0.15	0.96 (0.89; 1.04)	0.30
Frequency Missing = 1						
Have PHC services implemented specific protocols to combat COVID-19?						
No	-	-	-	-	-	-
Yes	0.96 (0.89; 1.04)	0.33	0.97 (0.91; 1.03)	0.30	1.01 (0.95; 1.06)	0.83
Have PHC healthcare professionals received any training to care for suspected cases of COVID-19 and manage users diagnosed with COVID-19?						
No	-	-	-	-	-	-
Yes	1.06 (0.97; 1.17)	0.21	1.04 (0.98; 1.1)	0.20	0.98 (0.9; 1.06)	0.58
Did healthcare professionals receive adequate PPE?						
No	-	-	-	-	-	-
Yes	0.97 (0.93; 1.02)	0.26	0.98 (0.94; 1.02)	0.25	1 (0.98; 1.02)	0.89
Were healthcare professionals provided with adequate quality PPE?						
No	-	-	-	-	-	-
Yes	1.02 (0.98; 1.05)	0.41	1 (0.98; 1.02)	1.00	0.98 (0.96; 1.01)	0.30
Were users of the health unit, when presenting flu-like symptoms with saturation levels lower than 95% during spontaneous breathing, in addition to presenting some comorbidity that puts them at risk for worsening COVID-19, referred to other RAS points?						
No	-	-	-	-	-	-
Yes	1.03 (0.98; 1.09)	0.20	1.01 (0.99; 1.04)	0.33	0.98 (0.93; 1.03)	0.38
Was information technology used for telecare in pre-clinical care, diagnostics, case monitoring and consultations?						
No	-	-	-	-	-	-
Yes	1.11 (0.91; 1.36)	0.30	1.11 (0.95; 1.3)	0.18	1 (0.85; 1.17)	0.99
Did users over 60 years of age, those with immunosuppression, chronic illnesses, pregnant women, and women in the postpartum period receive priority care when entering the health unit reporting signs and symptoms of flu-like illness?						
No	-	-	-	-	-	-
Yes	0.99 (0.95; 1.04)	0.74	0.99 (0.95; 1.02)	0.40	0.99 (0.96; 1.02)	0.64
Was monitoring of users considered at risk carried out via telephone every 24 hours?						
No	-	-	-	-	-	-
Yes	0.97 (0.83; 1.13)	0.68	0.87 (0.77; 0.98)	0.03	0.9 (0.81; 1)	0.05
Was monitoring of users with flu-like symptoms carried out via telephone every 48 hours?						
No	-	-	-	-	-	-
Yes	0.87 (0.76; 1)	0.04	0.87 (0.77; 0.97)	0.02	1 (0.92; 1.08)	0.92

*PHC - Primary Health Care; FHt - Family Health Team; PR - Prevalence Ratio; 95%CI - 95% Confidence Interval; p-value - significance level; RAS - Health Care Network; PPE - Personal Protective Equipment.*

The physical structure of PHC services underwent adaptations to address suspected and confirmed cases of COVID-19, with prevalence being 22% (CI: 0.66; 0.92) lower in municipalities with FHt coverage between 25% and 49.99% than in municipalities with coverage between 50% and 74.99%. This same variable was 27% (CI: 1.13; 1.42) more prevalent in municipalities with coverage between 50% and 74.99% than in municipalities with coverage greater than 75% (Table 2).

The notification of suspected/confirmed cases of COVID-19 within 24 hours was 1% (CI: 1; 1.02) more prevalent in municipalities with FHt coverage between 50% and 74.99% when compared to municipalities with coverage greater than 75%. Despite the result with a statistically significant difference, the epidemiological impact is small (Table 2).

Monitoring users considered at risk every 24 hours by telephone was 13% (CI: 0.77; 0.98) less prevalent in municipalities with FHt coverage between 25% and 49.99% when compared to municipalities with coverage greater than 75%. Furthermore, monitoring users with flu-like syndrome by telephone every 48 hours was 13% (CI: 0.76; 1) less prevalent in municipalities with FHt coverage between 25% and 49.99% when compared to municipalities with coverage between 50% and 74.99% and greater than 75% (Table 2).

## DISCUSSION

This study demonstrated that FHts were adapted to meet the demands arising from the COVID-19 pandemic and ensure routine care. However, each municipality reorganized services according to the characteristics of RAS in which it was located. Municipalities with lower FHt coverage stood out in the reorganization of service flows; this situation may be linked to the different organization of teams and the population size of these municipalities, since it is known that larger municipalities have lower FHt coverage and greater geographic proximity to services at all levels of care.

It is imperative to understand that municipalities with low and medium FHt coverage generally have a high Municipal Human Development Index (MHDI) and a high Gross Domestic Product (GDP) *per capita*, which can enhance individual efforts to address the health emergency. However, municipalities with greater FHt coverage tend to have lower MHDI and GDP *per capita*; however, they also have high FHt coverage^(14)^.

The greater FHt coverage in small municipalities may also be associated with the fact that, especially in remote locations, this may be the only type of healthcare service close to the population, ensuring the individual’s first access to the health system. In this scenario, it should be noted that the critical phase of the pandemic required a more effective and inclusive response from FHts to enhance their individual and collective actions, even if access to diagnostic tests and hospital care was limited^(15,16)^. Studies conducted in South Africa and Australia also demonstrated a strong capacity for restructuring and reorganizing PHC services during the critical period of the pandemic^(17,18)^.

Faced with an unknown, confusing, and challenging scenario that arose in the initial phase of the COVID-19 pandemic, especially in PHC, measures to implement effective actions for managing COVID-19 were urgently needed, including adapting the practice scenario, reorganizing the flow of care, and implementing technologies that ensured care and monitoring of diagnosed and suspected cases of the disease, in addition to redefining professionals’ work process^(19)^.

It is worth noting that, in the period leading up to the COVID-19 pandemic, community care, a principle of FHts, was partially neglected during the expansion phase of these services, experiencing significant gaps due to the limited and reduced number of community health workers. These gaps led to a slowdown in monitoring activities for the most vulnerable groups, such as children, pregnant women, older adults, and people with disabilities in the region. This scenario was compounded by setbacks in the Brazilian National Primary Care Policy, which severely impacted the organization of PHC throughout the country^(20-22)^.

In the pandemic scenario, specifically in the critical phase of the pandemic, it is worth noting that COVID-19 management and control were not directly related to a response and/or level of Fht coverage, since it refers to a rapidly spreading disease, and the health situation worsened rapidly, requiring readiness from emergency care and hospital services^(23)^. However, the coverage and reorganization of services provided by the FHt were one of the factors that positively impacted actions to combat COVID-19.

It is noteworthy that the extensive reach of FHts in PHC, especially in municipalities with above-average coverage, may be associated with the reduction in contagion and mortality rates from the disease during the initial phase of the pandemic. Thus, the valorization of PHC and FHt as the main services at this level of care in Brazil strengthens RAS, especially during health emergencies. Therefore, PHC services directly contribute to improving the population’s health status, especially the most socioeconomically vulnerable, as well as older adults, pregnant women, and children^(24)^.

A study showed that, despite the limitations and structural conditions, managers sought to reorganize PHC services in order to guarantee safe and adequate care^(25)^, highlighting the availability and training of healthcare professionals, appropriate physical structure to assist suspected cases of COVID-19, in addition to the availability of diagnostic tests in acceptable quantities, linked to the creation of adequate flows and protocols for access to healthcare services, epidemiological surveillance, appropriate and sufficient PPE^(26)^.

In Australia, New Zealand, Canada, the Netherlands, the United Kingdom, and the United States of America, during the period of mobility restrictions, there was a reduction in primary care services and continuity of services for conditions other than COVID-19. This situation is related to the emergency and priority imposed by the pandemic; in this context, the use of telehealth increased^(27)^.

In this context, remote activities such as teleconsultations should be promoted, as they provide and guarantee assistance during periods of physical distancing and mobility restrictions, favoring the continuity of people’s treatments^(28)^. To this end, managers must seek to implement technological infrastructure, digital health interventions, and ongoing training for healthcare professionals, as these have been considered effective elements in routine practices, especially in health emergencies, such as those experienced during the critical phase of COVID-19.

In Brazil, the way PHC is organized, with priority given to FHts, was considered the appropriate model to support people in facing measures to reduce and contain COVID-19^(19)^. This level of care should provide the majority of healthcare actions, in addition to strengthening the bond between professionals and users in the long term.

The onset of the pandemic, when the population experienced the presence of an unknown disease that spread rapidly throughout the world, causing a high number of deaths in a short period of time, with no effective medication and no vaccine, contributed to the focus of response actions during this phase on hospital care. Thus, PHC involvement in the response to the health crisis varied according to the organization of health systems in different countries. In countries such as China, India, and Cuba, PHC services were immediately reorganized. In Spain, PHC professionals were transferred to hospitals, interfering with the care provided within PHC. Therefore, it is important to emphasize that confronting a pandemic requires individual and community approaches, based on comprehensive care tailored to the population’s needs^(29)^.

The FHts’ performance during the critical years of the pandemic varied across Brazilian municipalities, taking into account their particularities and attributes, such as the coordination between healthcare services in RAS. Local peculiarities, such as population density, cultural and social aspects, the quality and resolution of PHC, and the coordination between RAS and other facilities, should have been respected during the various phases of the pandemic, requiring different local tactical capabilities at each specific moment during this period^(30)^.

It is understood that, in order to achieve one of the principles of the Brazilian Health System in PHC, resolution, in addition to professional training, professional commitment, availability of supplies and materials in adequate quantity and quality, as well as safe conditions for providing assistance and the concrete possibility of replacing professionals in exceptional cases are necessary, in order to protect their health^(31)^.

Furthermore, when inferring the effectiveness of FHts during the critical phase, it should be considered that, during this period, these professionals should have adequate access to tests for early diagnosis of the disease, in addition to being trained to make the necessary and timely referrals to other levels of care. In this context, the importance of integration with other levels of healthcare is highlighted, aiming to ensure qualified and comprehensive care throughout RAS^(29)^.

The existence of a strengthened PHC, broad FHt coverage and adequately structured, in addition to efficient management and adequate coordination capacity, provides a greater chance of achieving positive indicators in the face of a health emergency, ensuring facilitated and adequate access to the health system^(32)^.

Another challenge faced at the onset of the pandemic was the shortage of PPE. However, the lack of this equipment was felt in nursing homes and health units in the English context^(33)^. In this scenario, rich countries promptly guaranteed their internal supply of PPE, while simultaneously influencing the shortage in poorer countries dependent on external supply chains, in addition to a preferential direction for hospitals, rather than PHC services^(34)^. Greater availability of PPE in municipalities with lower FHt coverage may be related to municipal health management.

### Study limitations

This study presented as a limitation the low participation of managers from the North and Central-West regions as well as from Brazilian capitals. It is important to mention that the sample is not representative of all regions of the country, but this fact did not imply the robustness of results, since managers from all Brazilian states participated in the study.

### Contributions to public health

The broad participation of PHC managers in this study contributes to the reflection of the positive and negative aspects experienced during the pandemic, strengthening planning and management actions, with a view to meeting teams’ routine needs and also other health emergencies.

## CONCLUSIONS

In this study, when analyzing the adaptations made to PHC services to meet demand during the critical period of the pandemic, they were implemented with varying intensities and priorities, regardless of coverage level. Restructuring of PHC flow and referral and counter-referral services within RAS was predominant in municipalities with below-median FHt coverage. Adaptations to the physical structure of services to treat suspected and confirmed cases of COVID-19 and the notification of suspected/confirmed cases within 24 hours were more frequent in municipalities with median and high FHt coverage.

Specifically regarding the aforementioned changes, it is understood that municipalities with below-average coverage focused on reorganizing their flow, which may even be related to an extraterritorial increase in demand or even a reorganization of these services’ activities within RAS to serve the local population. On the other hand, municipalities with greater FHt coverage adapted their physical structures and acted more effectively in reporting cases. It can be inferred that these municipalities, because they already had greater availability of services and healthcare professionals, were able to adapt to meet the high demand.

The ongoing challenges in health management are undeniable, and they must focus on the population’s needs to facilitate timely responses during crises. Therefore, expanding and strengthening FHt as the primary PHC service is beneficial for both routine activities and future challenges.

## Data Availability

The research data are available within the article.
