# Supplementary material for: Managing COVID-19 care in the pandemic context: impact of family health coverage
Source: Rev Bras Enferm. 2025 Dec 8;78(6):e20240620. doi: 10.1590/0034-7167-2024-0620 (PMC12707611; doi:10.1590/0034-7167-2024-0620)
Supplement: Translation

# Translation: Gestão da assistência à COVID-19 no contexto pandêmico: impacto da cobertura da saúde da família

**DOI:** 10.1590/0034-7167-2024-0620pt

**Authors:** Ana Paula de Vechi Corrêa, Rodrigo das Neves Cano, Gustavo Diego Magno, Isabel María López-Medina, Carmen Álvarez-Nieto, Sílvia Carla da Silva André Uehara

**Affiliations:** I Universidade Federal de São Carlos. São Carlos, São Paulo, Brasil; II Universidad de Jaén. Jaén, Espanha

**Keywords:** COVID-19, Atenção Primária à, Saúde, Vigilância em Saúde Pública, Gestão em Saúde, Estratégias de Saúde Nacionais.

## Abstract

**Objetivos:**

comparar a gestão dos serviços de saúde da Atenção Primária à Saúde no enfrentamento da pandemia de COVID-19 entre os municípios brasileiros, considerando a cobertura das equipes de Saúde da Família.

**Métodos:**

estudo transversal descritivo, realizado com 1.134 gestores municipais de saúde. Os dados foram coletados no *Google Forms^®^*, analisados por Razões de Prevalência, utilizando modelo de regressão de Poisson com efeito aleatório.

**Resultados:**

municípios com cobertura das equipes menor que 25% apresentaram maior readaptação dos serviços e reestruturação do fluxo da Atenção Primária à Saúde (7%;IC:1;1,14), coberturas entre 25%, e 49,99% das equipes apresentaram maior reestruturação do fluxo da Atenção Primária à Saúde (7%;IC:1,02;1,12) e alterações dos serviços de referência e contrarreferência (21%;IC:1,05;1,38).

**Conclusões:**

as equipes reorganizaram e reestruturaram os serviços no período crítico da pandemia, fortalecendo a saúde da família como o principal serviço da Atenção Primária à Saúde.

## INTRODUÇÃO

A fase crítica da pandemia impôs, mundialmente, novos desafios aos sistemas de saúde devido à sua alta transmissibilidade, pouco conhecimento sobre tratamentos, e medidas eficazes de prevenção e medicamentos, além do início desafiador da vacinação com a insuficiência de imunobiológicos e sistema logístico despreparado em muitos países^(1)^.

Em um cenário sem precedentes na história moderna da saúde pública, a pandemia de COVID-19 exigiu uma adaptação ágil e eficaz nos sistemas de saúde, passando por obstáculos relacionados à alocação de recursos, gerenciamento de leitos, elaboração de protocolos, entre outros desafios. Nesse contexto, tornou-se essencial o trabalho dos gestores de saúde na coordenação dos serviços e na tomada de decisão com base em evidências e na implementação de medidas, com vistas à mitigação do impacto da doença na comunidade, além de buscarem manter a oferta de uma assistência de qualidade às condições crônicas e aos problemas comuns da Atenção Primária à Saúde (APS)^(2)^.

É inegável que a pandemia criou também oportunidades de aprendizado em diversos níveis de gestão, avultando a integração dos serviços de saúde e das Redes de Atenção à Saúde (RAS), coordenadas pela APS, além de aprimorar a cultura de gestão de risco, estabelecimento de ações educativas entre trabalhadores da saúde e comunidade, incorporação de tecnologias às rotinas do trabalho em saúde, e reconhecimento político, a fim de garantir recursos financeiros e fortalecer o sistema de saúde a médio e longo prazo^(3)^.

Um dos grandes desafios da APS, durante a pandemia, foi manter o acompanhamento de pessoas com doenças crônicas, aliado ao novo cenário imposto pela COVID-19 na reorganização das unidades de saúde e no próprio sistema de saúde local. Nos Estados Unidos da América, estudo revelou que os serviços de APS enfrentaram maiores desafios no acompanhamento de pacientes com doenças crônicas, destacando vulnerabilidades no sistema de saúde diante da ruptura dos processos de trabalho durante a pandemia^(4)^.

No Canadá, foi evidenciada a importância da integração entre a APS e RAS, com comunicação fluida entre as partes, com vistas à construção de um planejamento do cuidado, ao mesmo tempo que sugere a necessidade de alavancar um modelo de atenção direcionado à integração dos sistemas, provendo arranjos de governança que garantam essa integração^(5)^.

Além disso, a resposta à pandemia variou significativamente entre os países. Como exemplo disso, podem-se citar Coréia do Sul, Índia, Turquia e China, que optaram por uma abordagem com base na governança e forte intervenção estatal, enquanto outros, como Brasil, Portugal e Suíça, buscaram reforçar os modelos de atendimento preventivo e incorporaram a telemedicina^(6)^. Essas diferentes abordagens e experiências reforçam a importância da adaptação e aprendizado contínuo no enfrentamento de emergências sanitárias e na busca por soluções eficazes na prestação de cuidados de saúde à comunidade^(4)^.

Cabe destacar que, no Brasil, a Estratégia Saúde da Família (ESF) é a principal política da APS, desempenhando uma assistência à saúde com enfoque comunitário, e durante a fase crítica da pandemia, promoveu a reorganização dos serviços, de forma a garantir a promoção da qualidade de vida e a proteção da população assistida^(7)^. Ainda, a distribuição da ESF se apresenta de forma desigual no Brasil, apesar de apresentar uma cobertura da ESF de 62,6% dos brasileiros em 2019. Ressalta-se que as regiões Nordeste e Sul possuem as maiores coberturas de ESF. Por outro lado, a região Sudeste possui o maior número absoluto de brasileiros cadastrados, refletindo a maior concentração populacional^(8)^.

Contudo, faz-se necessário considerar que o Brasil, devido à sua extensão territorial, apresenta desigualdades nas condições de vida que condicionam as pessoas à exposição a riscos e agravos de saúde, bem como ao acesso aos serviços de saúde de forma desigual, além de apresentar uma maior concentração de bens e serviços em centros urbanos economicamente mais desenvolvidos^(9)^.

Tendo a fase crítica da pandemia de COVID-19 como ponto de partida, torna-se evidente que a atuação da APS foi afetada durante este período, exigindo um esforço e trabalho da gestão para a adequação desses serviços^(10)^. Entretanto, ainda permanece lacuna no conhecimento, especialmente no que se refere à forma como um país de dimensão continental reorganizou a APS para o enfrentamento da fase crítica da pandemia, considerando a gestão dos serviços em diferentes regiões do país e a influência da cobertura da equipes de Saúde da Família (eSF) nesse processo.

## OBJETIVOS

Comparar a gestão dos serviços de saúde da APS no enfrentamento da pandemia de COVID-19 entre os municípios brasileiros, considerando a cobertura da eSF.

## MÉTODOS

### Aspectos éticos

A pesquisa foi aprovada pelo Comitê de Ética em Pesquisa em Saúde da Universidade Federal de São Carlos. O Termo de Consentimento Livre e Esclarecido foi obtido de todos os indivíduos envolvidos no estudo por meio *online*.

### Desenho, período e local do estudo

Trata-se de estudo de corte transversal, descritivo, norteado pelo *STrengthening the Reporting of OBservational studies in Epidemiology*.

A população-alvo foi composta por gestores da APS dos municípios brasileiros que tiveram confirmado pelo menos um caso de COVID-12 no período de 26 de fevereiro de 2020 a 30 de junho de 2021. Foi realizado um cálculo amostral, considerando um gestor por município, sendo utilizado erro relativo de 5,82%, assumindo uma prevalência de 50% e um coeficiente de confiança de 95%. A amostra foi definida em 1.134 participantes.

Gestores da APS de um município por pelo menos três meses durante a fase de emergência sanitária imposta pela pandemia de COVID-19 foram incluídos para participar da pesquisa. Os responsáveis que durante o período da pandemia estavam de licença e/ou férias foram excluídos.

### Protocolo do estudo

Os dados foram coletados por meio de questionário auto-respondido pelos gestores de serviços da APS, por meio do *Google Forms^®^*, entre abril e setembro de 2022. Este questionário foi construído com base no protocolo de manejo clínico do coronavírus (COVID-19) na APS do Ministério da Saúde^(11)^, contendo as seguintes variáveis: identificação de caso suspeito de síndrome gripal e de COVID-19; medidas para evitar contágio nas unidades de saúde; estratificação da gravidade da síndrome gripal; manejo terapêutico e isolamento domiciliar de casos leves; diagnóstico precoce e encaminhamento a serviços de urgência/emergência ou hospitalares de casos graves; notificação imediata; monitoramento clínico; medidas de prevenção comunitária; e apoio à vigilância ativa.

Os questionários foram encaminhados por e-mail aos gestores municipais. Para aumentar a adesão, a pesquisa foi divulgada pelo Conselho Nacional de Secretários de Saúde e Conselho Nacional de Secretários Municipais de Saúde, que ressaltaram a importância da participação dos municípios e encaminharam o instrumento para as secretarias municipais de saúde. Os apoiadores dos Conselhos de Secretários Municipais de Saúde também colaboraram na divulgação da pesquisa junto aos Departamentos Regionais de Saúde.

### Análise dos resultados e estatística

Para a análise da cobertura da eSF, não foi encontrado na literatura um parâmetro ideal sobre a cobertura desses serviços. Assim, os pesquisadores definiram a seguinte classificação: < 25%; de 25,01% a 49,99%; de 50% a 74,99%; e >75%. A cobertura da eSF dos municípios participantes foi retirada do site e-Gestor AB do Ministério da Saúde^(12)^.

Frequências absolutas e relativas foram utilizadas para descrever as variáveis qualitativas. Para estimar as Razões de Prevalência, comparando faixa de cobertura da eSF, foi utilizado o modelo de regressão de Poisson com efeito aleatório^(13)^. Todas as análises foram realizadas por meio do *software* SAS 9.4. Para todas as análises, adotou-se um nível de significância de 5%.

## RESULTADOS

Participaram da pesquisa 1.134 gestores procedentes de todo o país, sendo 40,4% (458) do Sudeste, 27,9% (316), do Nordeste, 18,6% (211), do Sul, 8,5% (97), do Norte e 4,6% (52), do Centro-Oeste. Desses, 79,4% (900) referiram ser do sexo feminino e 20,6% (234), do sexo masculino, com uma média de 39,7 anos. Sobre a cobertura da eSF, destaca-se que a cobertura maior que 75% estava presente em 896 (79%) municípios participantes.

Nas comparações entre a cobertura da eSF e a reorganização dos serviços da APS no enfrentamento da pandemia, todos os participantes responderam que os serviços da APS foram adaptados para o enfrentamento da COVID-19. Nesse contexto, evidenciou-se que a prevalência da readaptação dos serviços e reestruturação do fluxo da APS foi 7% (IC: 1; 1,14) maior em municípios com cobertura da eSF menor que 25%, quando comparados aos municípios com cobertura maior que 75%. Além disso, a notificação dos casos suspeitos/confirmados de COVID-19 em até 24 horas foi 1% (1; 1,02) maior em municípios com cobertura da eSF menor que 25%, quando comparados aos municípios com cobertura maior que 75% (Tabela 1).

**Tabela 1:** Organização da gestão da Atenção Primária à Saúde no enfrentamento da pandemia de COVID-19, considerando a cobertura da equipe de Saúde da Família <25% *versus* **entre 25% e 49,99%; <25%** *versus* **entre 50% e 74,99%; e <25%** *versus* >75%

Variável	<25% *versus* entre 25% e 49,99%		<25% *versus* entre 50% e 74,99%		<25% *versus* ≥75%	
	RP (IC95%)	Valor de *p*	RP (IC95%)	Valor de *p*	RP (IC95%)	Valor de *p*
Os serviços da APS foram adaptados para o enfrentamento da COVID-19?						
Não	-	-	-	-	-	-
Sim	1,03 (0,96; 1,11)	0,43	0,99 (0,93; 1,05)	0,72	1,07 (1; 1,14)	0,04
O fluxo da APS foi reestruturado durante a pandemia de COVID-19?						
Não	-	-	-	-	-	-
Sim	1,01 (0,94; 1,08)	0,88	1,03 (0,96; 1,1)	0,48	1,07 (1,01; 1,14)	0,02
Os serviços de referência e contrarreferência da APS dentro da RAS sofreram alterações durante a pandemia de COVID-19?						
Não	-	-	-	-	-	-
Sim	1,03 (0,79; 1,33)	0,85	1,04 (0,79; 1,38)	0,76	1,24 (0,97; 1,58)	0,09
*Frequency Missing* = 1						
A estrutura física dos serviços da APS sofreu adequações para atender os casos suspeitos e confirmados de COVID-19?						
Não	-	-	-	-	-	-
Sim	1,16 (0,87; 1,53)	0,31	0,9 (0,69; 1,18)	0,46	1,15 (0,89; 1,48)	0,30
Os profissionais de saúde considerados de risco para o desenvolvimento da forma grave de COVID-19 foram alocados para *home office*?						
Não	-	-	-	-	-	-
Sim	0,82 (0,65; 1,03)	0,08	0,8 (0,64; 1)	0,05	0,8 (0,64; 1)	0,05
Os casos suspeitos de COVID-19, ao serem recebidos pelas unidades de saúde, são notificados pelos profissionais de saúde em até 24 horas?						
Não	-	-	-	-	-	-
Sim	1,01 (0,99; 1,03)	0,33	1 (1; 1)	,	1,01 (1; 1,02)	<0,01
Os casos de COVID-19 notificados na RAS são avaliados previamente pela APS?						
Não	-	-	-	-	-	-
Sim	1,05 (0,88; 1,24)	0,61	1 (0,87; 1,15)	0,97	0,96 (0,85; 1,08)	0,50
*Frequency Missing* = 1						
Os serviços da APS implementaram protocolos específicos para o enfrentamento da COVID-19?						
Não	-	-	-	-	-	-
Sim	1,04 (0,93; 1,15)	0,51	1 (0,9; 1,1)	0,92	1 (0,92; 1,09)	0,98
Os profissionais de saúde da APS receberam alguma capacitação para o atendimento de casos suspeitos de COVID-19 e manejo de usuários com diagnóstico de COVID-19?						
Não	-	-	-	-	-	-
Sim	1,01 (0,92; 1,12)	0,83	1,07 (0,96; 1,21)	0,22	1,05 (0,96; 1,15)	0,28
Os profissionais de saúde receberam EPIs em quantidade adequada?						
Não	-	-	-	-	-	-
Sim	1,03 (0,99; 1,08)	0,10	1,01 (0,99; 1,03)	0,31	1,01 (1; 1,02)	<0,01
Os profissionais de saúde receberam EPIs de qualidade adequada?						
Não	-	-	-	-	-	-
Sim	1,01 (0,99; 1,03)	0,33	1,03 (1; 1,06)	0,06	1,01 (1; 1,02)	<0,01
Os usuários da unidade de saúde, ao apresentarem síndrome gripal com saturação menor que 95% em respiração espontânea, além de apresentarem alguma comorbidade de risco para o agravamento da COVID-19, foram encaminhados a outros pontos da RAS?						
Não	-	-	-	-	-	-
Sim	0,92 (0,83; 1,03)	0,14	0,96 (0,85; 1,07)	0,44	0,94 (0,84; 1,04)	0,21
Houve utilização de tecnologias da informação para o teleatendimento em atendimentos pré-clínicos, diagnósticos, acompanhamento dos casos e consultas?						
Não	-	-	-	-	-	-
Sim	0,92 (0,7; 1,22)	0,58	1,03 (0,78; 1,37)	0,85	1,03 (0,8; 1,32)	0,83
Usuários com faixa etária maior que 60 anos, portadores de imunossupressão, doentes crônicos, gestantes e mulheres que estão em fase de puerpério, ao entrarem na unidade de saúde relatando sinais e sintomas de síndrome gripal, receberam atendimento prioritário?						
Não	-	-	-	-	-	-
Sim	1,03 (1; 1,07)	0,05	1,03 (1; 1,06)	0,08	1,02 (1,01; 1,03)	<0,01
O acompanhamento dos usuários considerados de risco foi realizado via telefone a cada 24 horas?						
Não	-	-	-	-	-	-
Sim	1,01 (0,81; 1,25)	0,93	0,98 (0,79; 1,2)	0,83	0,88 (0,73; 1,05)	0,17
O acompanhamento dos usuários com síndrome gripal foi realizado via telefone a cada 48 horas?						
Não	-	-	-	-	-	-
Sim	0,93 (0,75; 1,17)	0,55	0,81 (0,66; 1)	0,05	0,81 (0,67; 0,99)	0,04

*Nota: APS - Atenção Primária à Saúde; eSF - equipe de Saúde da Família; RP - Razão de Prevalência; IC95% - Intervalo de Confiança de 95%; Valor de p - nível de significância; RAS - Rede de Atenção à Saude; EPIs - Equipamentos de Proteção Individual.*

Em relação aos Equipamentos de Proteção Individual (EPIs), a disponibilidade em quantidade e qualidade adequadas de EPIs foi 1% (IC: 1; 1,02) mais prevalente em municípios com cobertura eSF menor que 25%, quando comparados aos municípios com cobertura maior que 75%. Fato semelhante foi encontrado na priorização dos atendimentos dos grupos de risco para agravamento da COVID-19, que também foi 2% (IC: 1,01; 1,03) mais prevalente em municípios com cobertura da eSF menor que 25%, quando comparados aos municípios com cobertura maior que 75% (Tabela 1).

É importante destacar que, apesar dos resultados terem apresentado diferença estatística significante, quando avaliados na prática, esses valores de 1% e 2% não trazem impacto epidemiológico.

Já o acompanhamento dos pacientes com COVID-19 por meio telefônico a cada 48 horas foi 19% (IC: 0,67; 0,99) menos prevalente em municípios com cobertura da eSF menor que 25%, quando comparados aos municípios com cobertura maior que 75% (Tabela 1).

Em municípios com cobertura entre 50% e 74,99% da eSF, a adaptação dos serviços da APS para o enfrentamento da pandemia foi 8% (IC: 1,04; 1,13) mais prevalente do que naqueles com cobertura maior que 75%. Para a reestruturação do fluxo da APS durante a pandemia, a prevalência foi 7% (IC: 1,02; 1,12) maior em municípios com cobertura da eSF entre 25% e 49,99% do que naqueles com cobertura maior que 75%. As alterações nos serviços de referência e contrarreferência da APS dentro da RAS foram 21% (IC: 1,05; 1,38) mais prevalentes em municípios com cobertura da eSF entre 25% e 49,99%, quando comparadas aos municípios com coberturas maiores que 75% (Tabela 2).

**Tabela 2:** Organização da gestão da Atenção Primária à Saúde no enfrentamento da pandemia de COVID-19, considerando a cobertura da equipe de Saúde da Família entre 25% e 49,99% *versus* **entre 50% e 74,99%; entre 25% e 49,99%** *versus* >**75%; entre 50% e 74,99%** *versus* >75%

Variável	Entre 25% e 49,99% *versus* entre 50% e 74,99%		Entre 25% e 49,99% *versus* ≥75%		Entre 50% e 74,99% *versus* ≥75%	
	RP (IC95%)	Valor de *p*	RP (IC95%)	Valor de *p*	RP (IC95%)	Valor de *p*
Os serviços da APS foram adaptados para o enfrentamento da COVID-19?						
Não	-	-	-	-	-	-
Sim	0,96 (0,91; 1,01)	0,11	1,04 (0,99; 1,1)	0,14	1,08 (1,04; 1,13)	<0,01
O fluxo da APS foi reestruturado durante a pandemia de COVID-19?						
Não	-	-	-	-	-	-
Sim	1,02 (0,96; 1,08)	0,51	1,07 (1,02; 1,12)	<0,01	1,05 (0,99; 1,1)	0,08
Os serviços de referência e contrarreferência da APS dentro da RAS sofreram alterações durante a pandemia de COVID-19?						
Não	-	-	-	-	-	-
Sim	1,02 (0,84; 1,23)	0,85	1,21 (1,05; 1,38)	<0,01	1,18 (1; 1,4)	0,05
*Frequency Missing* = 1						
A estrutura física dos serviços da APS sofreu adequações para atender os casos suspeitos e confirmados de COVID-19?						
Não	-	-	-	-	-	-
Sim	0,78 (0,66; 0,92)	<0,01	0,99 (0,85; 1,15)	0,92	1,27 (1,13; 1,42)	<0,01
Os profissionais de saúde considerados de risco para o desenvolvimento da forma grave de COVID-19 foram alocados para *home office*?						
Não	-	-	-	-	-	-
Sim	0,98 (0,88; 1,09)	0,69	0,98 (0,91; 1,07)	0,70	1 (0,94; 1,08)	0,90
Os casos suspeitos de COVID-19, ao serem recebidos pelas unidades de saúde, são notificados pelos profissionais de saúde em até 24 horas?						
Não	-	-	-	-	-	-
Sim	0,99 (0,97; 1,01)	0,33	1 (0,98; 1,02)	1,00	1,01 (1; 1,02)	<0,01
Os casos de COVID-19 notificados na RAS são avaliados previamente pela APS?						
Não	-	-	-	-	-	-
Sim	0,95 (0,83; 1,09)	0,50	0,92 (0,81; 1,03)	0,15	0,96 (0,89; 1,04)	0,30
*Frequency Missing* = 1						
Os serviços da APS implementaram protocolos específicos para o enfrentamento da COVID-19?						
Não	-	-	-	-	-	-
Sim	0,96 (0,89; 1,04)	0,33	0,97 (0,91; 1,03)	0,30	1,01 (0,95; 1,06)	0,83
Os profissionais de saúde da APS receberam alguma capacitação para o atendimento de casos suspeitos de COVID-19 e manejo de usuários com diagnóstico de COVID-19?						
Não	-	-	-	-	-	-
Sim	1,06 (0,97; 1,17)	0,21	1,04 (0,98; 1,1)	0,20	0,98 (0,9; 1,06)	0,58
Os profissionais de saúde receberam EPIs em quantidade adequada?						
Não	-	-	-	-	-	-
Sim	0,97 (0,93; 1,02)	0,26	0,98 (0,94; 1,02)	0,25	1 (0,98; 1,02)	0,89
Os profissionais de saúde receberam EPIs de qualidade adequada?						
Não	-	-	-	-	-	-
Sim	1,02 (0,98; 1,05)	0,41	1 (0,98; 1,02)	1,00	0,98 (0,96; 1,01)	0,30
Os usuários da unidade de saúde, ao apresentarem síndrome gripal com saturação menor que 95% em respiração espontânea, além de apresentarem alguma comorbidade de risco para o agravamento da COVID-19, foram encaminhados a outros pontos da RAS?						
Não	-	-	-	-	-	-
Sim	1,03 (0,98; 1,09)	0,20	1,01 (0,99; 1,04)	0,33	0,98 (0,93; 1,03)	0,38
Houve utilização de tecnologias da informação para o teleatendimento em atendimentos pré-clínicos, diagnósticos, acompanhamento dos casos e consultas?						
Não	-	-	-	-	-	-
Sim	1,11 (0,91; 1,36)	0,30	1,11 (0,95; 1,3)	0,18	1 (0,85; 1,17)	0,99
Usuários com faixa etária maior que 60 anos, portadores de imunossupressão, doentes crônicos, gestantes e mulheres que estão em fase de puerpério, ao entrarem na unidade de saúde relatando sinais e sintomas de síndrome gripal, receberam atendimento prioritário?						
Não	-	-	-	-	-	-
Sim	0,99 (0,95; 1,04)	0,74	0,99 (0,95; 1,02)	0,40	0,99 (0,96; 1,02)	0,64
O acompanhamento dos usuários considerados de risco foi realizado via telefone a cada 24 horas?						
Não	-	-	-	-	-	-
Sim	0,97 (0,83; 1,13)	0,68	0,87 (0,77; 0,98)	0,03	0,9 (0,81; 1)	0,05
O acompanhamento dos usuários com síndrome gripal foi realizado via telefone a cada 48 horas?						
Não	-	-	-	-	-	-
Sim	0,87 (0,76; 1)	0,04	0,87 (0,77; 0,97)	0,02	1 (0,92; 1,08)	0,92

*APS - Atenção Primária à Saúde; eSF - equipe de Saúde da Família; RP - Razão de Prevalência; IC95% - Intervalo de Confiança de 95%; Valor de p - nível de significância; RAS - Rede de Atenção à Saude; EPIs - Equipamentos de Proteção Individual.*

A estrutura física dos serviços da APS sofreu adequações para atender aos casos suspeitos e confirmados de COVID-19, sendo 22% (IC: 0,66; 0,92) menos prevalente em municípios com cobertura da eSF entre 25% e 49,99% do que em municípios com coberturas entre 50% e 74,99%. Essa mesma variável foi 27% (IC: 1,13; 1,42) mais prevalente em municípios com coberturas entre 50% e 74,99% do que em municípios com coberturas maiores que 75% (Tabela 2).

A notificação dos casos suspeitos/confirmados de COVID-19 em até 24 horas foi 1% (IC: 1; 1,02) mais prevalente em municípios com cobertura da eSF entre 50% e 74,99%, quando comparados aos municípios com cobertura maior que 75%. Apesar do resultado com diferença estatística significante, o impacto epidemiológico é pequeno (Tabela 2).

O acompanhamento de usuários considerados de risco a cada 24 horas por telefone foi 13% (IC: 0,77; 0,98) menos prevalente em municípios com cobertura da eSF entre 25% e 49,99%, quando comparados aos municípios com cobertura maior que 75%. Além disso, o acompanhamento de usuários com síndrome gripal via telefone a cada 48 horas foi 13% (IC: 0,76; 1) menos prevalente em municípios com cobertura da eSF entre 25% e 49,99%, quando comparados aos municípios com cobertura entre 50% e 74,99% e maior que 75% (Tabela 2).

## DISCUSSÃO

Neste estudo, foi evidenciado que as eSFs foram adaptadas para atender às demandas oriundas da pandemia de COVID-19, bem como garantir os atendimentos de rotina, mas cada município reorganizou os serviços conforme as características da RAS em que estava inserido. Municípios com menor cobertura da eSF se destacaram na reorganização dos fluxos de atendimentos; tal situação pode estar atrelada às diferentes formas de organização das equipes e ao porte populacional desses municípios, uma vez que é sabido que municípios maiores possuem menor cobertura da eSF e uma maior proximidade geográfica com os serviços de todos os níveis de assistência.

Torna-se imperativo analisar que municípios com coberturas baixa e média da eSF geralmente apresentam Índice de Desenvolvimento Humano Municipal (IDHM) alto e Produto Interno Bruto (PIB) *per capita* elevado, o que pode potencializar ações de enfrentamento da emergência sanitária na dimensão individual. Entretanto, municípios com uma maior cobertura da eSF tendem a apresentar IDHM e PIB *per capita* mais baixos; contudo, possuem uma elevada cobertura da eSF^(14)^.

A maior cobertura da eSF em municípios pequenos também pode estar associada ao fato de que, especialmente, em localidades remotas, esse pode ser o único tipo de serviço de saúde próximo à população, de forma a garantir o primeiro acesso do individuo ao sistema de saúde. Nesse cenário, deve-se destacar que a fase crítica da pandemia exigiu uma resposta mais efetiva e inclusiva das eSFs, de forma a potencializar as suas ações individuais e coletivas, mesmo que o acesso a testes diagnósticos e à atenção hospitalar fosse limitado^(15,16)^. Estudos realizados na África do Sul e na Austrália também evidenciaram uma forte capacidade de reestruturação e reorganização dos serviços da APS no período crítico da pandemia^(17,18)^.

Diante de um cenário desconhecido, confuso e desafiador que se impôs na fase inicial da pandemia de COVID-19, especialmente na APS, foram urgentes as medidas que implementassem ações efetivas para o manejo da COVID-19, incluindo adaptação do cenário de prática, reorganização do fluxo de atendimento e implementação de tecnologias que garantissem a assistência e monitoramento dos casos diagnosticados e suspeitos da doença, além da redefinição do processo de trabalho dos profissionais^(19)^.

Nota-se que, no período que antecedeu à pandemia de COVID-19, o cuidado comunitário, princípio da eSF, foi em parte negligenciado na fase de expansão desses serviços, vivenciando fissuras importantes diante da limitação e redução dos agentes comunitários de saúde. Essas fissuras implicaram o arrefecimento de atividades de acompanhamento dos grupos mais vulneráveis, como crianças, gestantes, idosos e pessoas com deficiência no território. A esse cenário, também se adicionaram retrocessos na Política Nacional de Atenção Básica que impactaram intensamente a organização da APS em todo país^(20-22)^.

Já no cenário pandêmico, especificamente na fase crítica da pandemia, vale ressaltar que o manejo e controle da COVID-19 não estavam relacionados diretamente a uma resposta e/ou nível de cobertura da eSF, uma vez que se refere a uma doença de disseminação rápida, e o quadro de saúde piorava rapidamente, exigindo prontidão dos serviços de pronto atendimento e hospitalares^(23)^. Entretanto, a cobertura e reorganização dos serviços prestados pelas eSF se apresentaram como um dos fatores que impactaram positivamente as ações de enfrentamento da COVID-19.

Ressalta-se que a extensa capilaridade da eSF na APS, especialmente em municípios com coberturas acima da média, pode estar associada à redução das taxas de contágio e mortalidade pela doença durante a fase inicial da pandemia. Assim, a valorização da APS e das eSF como os principais serviços desse nível de assistência no Brasil proporciona um fortalecimento da RAS, especialmente em emergências sanitárias. Para tanto, os serviços da APS contribuem diretamente para a alteração dos níveis de saúde da população, especialmente as mais vulneráveis socioeconomicamente, além de idosos, gestantes e crianças^(24)^.

Estudo evidenciou que, apesar das limitações e condições estruturais, gestores buscaram reorganizar os serviços da APS, a fim de garantir uma assistência segura e adequada^(25)^, destacando-se a disponibilidade e capacitação de profissionais de saúde, estrutura física apropriada para assistir casos suspeitos de COVID-19, além da disponibilidade de testes diagnósticos em quantidade aceitável, atrelados à criação de fluxos e protocolos adequados para acesso aos serviços de saúde, vigilância epidemiológica, EPIs apropriados e suficientes^(26)^.

Na Austrália, Nova Zelândia, Canadá, Holanda, Reino Unido e Estados Unidos da América, durante o período de restrição de mobilidade, houve redução de atendimentos e continuidade nos serviços de saúde da APS, para condições além da COVID-19. Tal situação está relacionada ao fato da emergência e prioridade impostas pela pandemia; neste cenário, ampliou-se a utilização de telessaúde^(27)^.

Nesse contexto, atividades remotas como a teleconsulta devem ser impulsionadas, uma vez que proporcionam e garantem assistência em períodos de distanciamento físico e restrição de mobilidade, favorecendo a continuidade de tratamentos das pessoas^(28)^. Para tanto, gestores devem buscar implementar estrutura tecnológica, intervenções de saúde digital, além de capacitação contínua dos profissionais de saúde, uma vez que têm sido considerados elementos efetivos na rotina, mas principalmente em emergências sanitárias, como a vivenciada durante a fase crítica da COVID-19.

No Brasil, a forma de organização da APS, com prioridade para a eSF, foi considerada o modelo apropriado para amparar as pessoas no enfrentamento das medidas de redução e contenção da COVID-19^(19)^. Esse nível de assistência deve ofertar a maioria das ações de saúde, além propiciar o fortalecimento do vínculo entre profissionais e usuários a longo prazo.

O início da pandemia, em que a população vivenciava a presença de uma doença desconhecida que se disseminou rapidamente pelo mundo, causando elevado número de óbitos em curto tempo e sem nenhum medicamento eficaz e ausência de vacina, contribuiu para que as ações de enfrentamento utilizadas nesta fase fossem direcionadas para o atendimento hospitalar. Assim, a implicação da APS nas respostas direcionadas à crise sanitária variou de acordo com a organização dos sistemas de saúde nos diferentes países. Em países como China, Índia e Cuba, os serviços da APS foram reorganizados imediatamente. Já na Espanha, profissionais da APS foram transferidos para hospitais, interferindo na assistência ofertada na APS. Para tanto, cabe destacar que o enfrentamento de uma pandemia exige abordagens individuais e comunitárias, com base no cuidado integral e articulado às necessidades populacionais^(29)^.

O desempenho das eSFs nos anos críticos da pandemia ocorreu de maneira diferenciada nos municípios brasileiros, considerando as particularidades e atributos próprios, como a articulação entre os serviços de saúde na RAS. As peculiaridades locais, como densidade demográfica, aspectos culturais e sociais, qualidade e resolutividade da APS, além da articulação entre RAS e outros equipamentos, deveriam ter sido respeitados durante as diversas fases da pandemia, de forma a exigir diferentes capacidades táticas locais em cada momento específico desse período^(30)^.

Entende-se que, para o alcance de um dos princípios do Sistema Único de Saúde na APS, a resolutividade, além da capacitação profissional, são necessários o comprometimento dos profissionais, disponibilidade de insumos e materiais em quantidade e qualidade adequadas, bem como condições seguras para a realização da assistência e possibilidade concreta de substituição de profissionais em casos excepcionais, de forma a proteger a saúde dos mesmos^(31)^.

Ainda, ao inferir sobre a resolutividade das eSFs durante a fase crítica, deve-se considerar que, nesse período, esses profissionais deveriam ter acesso em quantidade adequada aos testes para realizar diagnóstico precoce da doença, além de estarem capacitados para realizar os encaminhamentos necessário e em tempo hábil para outros níveis de assistência. Nesse contexto, destaca-se a importância da integração com os demais níveis de assistência à saúde, com vistas a garantir um cuidado qualificado e integral em toda a RAS^(29)^.

A existência de uma APS fortalecida, ampla cobertura da eSF e adequadamente estruturada, além de uma gestão eficiente e adequada capacidade de coordenação, proporciona maior chance de alcançar indicadores positivos diante de uma emergência sanitária, garantindo acesso facilitado e adequado ao sistema de saúde^(32)^.

Outro desafio enfrentado o início da pandemia foi a escassez de EPIs. Contudo, a ausência desses equipamentos foi sentida em casas de repouso e unidades de saúde no contexto inglês^(33)^. Nesse cenário, países ricos garantiram prontamente seu provimento interno de EPIs, ao mesmo tempo que influenciaram a escassez para países mais pobres e dependentes de redes de suprimentos externas, além de um direcionamento preferencial para hospitais, em detrimento dos serviços da APS^(34)^. Maior disponibilidade de EPIs em municípios com uma menor cobertura da eSF pode estar relacionado à gestão municipal da saúde.

### Limitações do estudo

Este estudo apresentou como limitação a baixa adesão dos gestores das regiões Norte e Centro-Oeste, assim como das capitais brasileiras, sendo importante mencionar que a amostra não é representativa de todas as regiões do país, mas este fato não implicou a robustez dos resultados, uma vez que gestores de todos os estados brasileiros participaram do estudo.

### Contribuições para a área da saúde pública

A ampla participação dos gestores da APS neste estudo contribui para a reflexão dos aspectos positivos e negativos vivenciados durante a pandemia, fortalecendo as ações de planejamento e gestão das atividades, com vistas às necessidades de rotina das equipes e também às outras emergências de saúde.

## CONCLUSÕES

Neste estudo, ao analisar as adaptações realizadas nos serviços da APS para atender às demandas durante o período crítico da pandemia, foram realizadas com intensidades e prioridades diferentes, independentemente do nível de cobertura. A reestruturação do fluxo da APS e dos serviços de referência e contrarreferência dentro da RAS foi predominante nos municípios com cobertura de eSF inferior à mediana. Já adequações na estrutura física dos serviços para atender casos suspeitos e confirmados de COVID-19 e a notificação dos casos suspeitos/confirmados em até 24 horas foram mais frequentes em municípios com coberturas medianas e alta da eSF.

Especificamente para as alterações supracitadas, entende-se que municípios com uma cobertura inferior à média se preocuparam em reorganizar o fluxo, o que pode estar relacionado até mesmo a um aumento extraterritorial da demanda ou mesmo a uma reorganização das atividades desses serviços dentro da RAS para atender à população local. Por outro lado, municípios com uma maior cobertura da eSF adequaram a estrutura física e atuaram de forma mais efetiva na notificação dos casos, podendo inferir que esses municípios, por já terem uma maior disponibilidade de serviços e profissionais de saúde, conseguiram se adequar para atender à elevada demanda.

São inegáveis os desafios permanentes na gestão da saúde, que devem focar nas necessidades da população para facilitar respostas oportunas durante as crises. Desta forma, a ampliação e fortalecimento das eSF como o principal serviço da APS se tornam favoráveis para as atividades de rotina e também para os desafios futuros.

## Data Availability

Os dados da pesquisa estão disponíveis no corpo do artigo.
